# Analysis artefacts of the INS-IGF2 fusion transcript

**DOI:** 10.1186/s12867-015-0042-8

**Published:** 2015-07-29

**Authors:** Rasmus Wernersson, Thomas Frogne, Claude Rescan, Lena Hansson, Christine Bruun, Mads Grønborg, Jan Nygaard Jensen, Ole Dragsbæk Madsen

**Affiliations:** Intomics A/S, Diplomvej 377, 2800 Lyngby, Denmark; Center for Biological Sequence Analysis, Technical University of Denmark, 2800 Lyngby, Denmark; Novo Nordisk A/S, Novo Nordisk Park, 2760 Måløv, Denmark

**Keywords:** Insulin, INS-IGF2, Beta cell, Fusion-transcript, Gene expression, Proteomics, Antibody, Bioinformatics, Analysis artefact, Pancreatic islets, Diabetes

## Abstract

**Background:**

In gene expression analysis, overlapping genes, splice variants, and fusion transcripts are potential sources of data analysis artefacts, depending on how the observed intensity is assigned to one, or more genes. We here exemplify this by an in-depth analysis of the INS-IGF2 fusion transcript, which has recently been reported to be among the highest expressed transcripts in human pancreatic beta cells and its protein indicated as a novel autoantigen in Type 1 Diabetes.

**Results:**

Through RNA sequencing and variant specific qPCR analyses we demonstrate that the true abundance of INS-IGF2 is >20,000 fold lower than INS in human beta cells, and we suggest an explanation to the nature of the artefacts which have previously led to overestimation of the gene expression level in selected studies. We reinvestigated the previous reported findings of detection of INS-IGF2 using antibodies both in Western blotting and immunohistochemistry. We found that the one available commercial antibody (BO1P) raised against recombinant INS-IGF2 show strong cross-reaction to native proinsulin, and we did not detect INS-IGF2 protein in the human beta cell line EndoC-βH1. Furthermore, using highly sensitive proteomics analysis we could not demonstrate INS-IGF2 protein in samples of human islets nor in EndoC-βH1.

**Conclusions:**

Sequence features, such as fusion transcripts spanning multiple genes can lead to unexpected results in gene expression analysis, and care must be taken in generating and interpreting the results. For the specific case of INS-IGF2 we conclude that the abundance of the fusion transcript/protein is exceedingly lower than previously reported, and that current immuno-reagents available for detecting INS-IGF2 protein have a strong cross-reaction to native human proinsulin. Finally, we were unable to detect INS-IGF2 protein by proteomics analysis.

**Electronic supplementary material:**

The online version of this article (doi:10.1186/s12867-015-0042-8) contains supplementary material, which is available to authorized users.

## Background

### Gene expression analysis artefacts

**Fig. 1:**
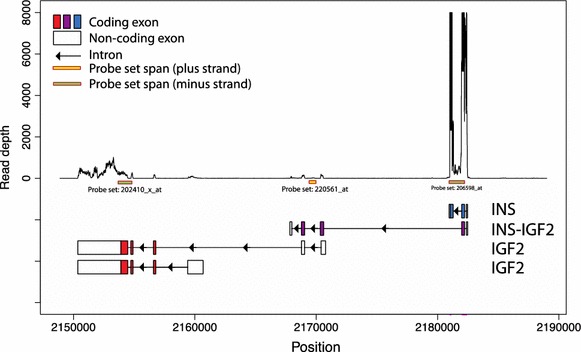
RNA sequencing read depth in the INS/IGF2 genomic region. Plot of the genomic region of chromosome 11 around the INS and IGF2 loci (2150,000–2190,000; genomic assembly: GRCh37/hg19) with probesets from the Affymetix HG-U133A array and the main transcript variants of INS, INS-IGF2 and IGF2 indicated below the plot. All shown transcripts are encoded on the antisense strand (as indicated by the direction of the* arrows* in the introns)—for an overview of all known transcript variants, please see Additional file 1: Figure S1 (panel A). The y-axis shows the read depth of the RNA sequencing study from Nica et al. [1]—notice that read depth was capped at 8,000 (data shown for beta cell dataset #6—the same pattern was observed for all other data sets). From the plot it is clearly seen that few reads maps to the INS-IGF2 exons in the 2170000 area (middle of the plot) and almost all signal assigned to INS-IGF2 by Nica et al. [1] originates from the first coding exon which is shared with INS (*right hand* *side* of the plot). All positional information on this plot is from ENSEMBL v.75.

As part of our research on pancreatic beta cell biology, our group has been investigating the transcripts originating from the insulin loci using both microarrays and RNA-sequencing platforms. On several occasions we have noticed issues with the way overlapping transcripts are handled in gene expression analysis pipelines, and this sparked the interest for looking further into the underlying reasons for the problems. Our initial observations were as follows:

In DNA microarray analysis different results will be obtained for the genes in the genomic region around INS (Ensembl: ENSG00000254647) depending on the mapping scheme used. In extreme cases INS itself will disappear from the analysis and all its signal would instead be mapped to INS-IGF2 (Ensembl: ENSG00000129965). The reason for this artefact turned out to be that certain widely used enhanced remapping schemes for Affymetrix probes (including the popular BrainArray CDF files) are targeting the longest transcript in the region. In most cases this will not cause an issue, since all the transcripts variants will in the end be mapped to the same underlying gene. However, in the case of INS the issue is that a fusion transcript, INS-IGF2, is spanning the entire region (see Fig. 1 and Additional file 1: Figure S1), including both insulin exons as well as part of the adjacent IGF2-locus. This will lead to all signal in the region being mapped to INS-IGF2 and that INS will “disappear” from the analysis, and in certain cases so would IGF2 (Ensembl: ENSG00000167244). This will happen even if there are no probes targeting the unique part of the fusion transcript.

For RNA sequencing analysis a related problem is encountered. The individual reads will typically be mapped to a set of transcripts from the organism in question, and will be assigned multiple times to transcripts sharing sequences. In most cases this is the desired behaviour, and will be a way to handle splicing variants of a given gene (and a more thorough analysis of exon/exon junctions can be investigated). However, in the case of transcripts spanning several genes, there is a risk that the signal from one gene bleeds into the overlapping transcript, and this may not be apparent when the results are reported.

With this in mind, we decided to re-investigate the observation by Nica et al. [1] that the INS-IGF2 fusion transcript was found to be the second most abundant mRNA in human beta cells, an observation that has generated much interest in the beta cell biology field since INS-IGF2 has previously been annotated to be translated [2] and moreover, recently proposed as a novel autoantigen in Type 1 Diabetes (T1D) [3].

### Case background: beta cell biology

The pancreatic beta cell is a key component in regulating blood glucose homeostasis as it represents our sole source of insulin, which is required for peripheral tissues to internalize and utilize glucose as an energy source. Lack of adequate functional beta cell mass leading to relative insulin insufficiency is the common denominator of all forms of diabetes. T1D is characterized by an absolute loss of beta cells as a result of autoimmune destruction. Type 2 diabetes (T2D) is where a relative beta cell insufficiency results from failure of compensatory upregulation of insulin production capacity in response to increasing peripheral insulin resistance. Therefore much effort in, both preventive and curative, diabetes research has aimed at understanding the molecular networks, both at the level of transcriptional and translational regulation of genes controlling development and at the function and survival of the pancreatic beta cell. Recent advancements in the ability to purify single cell types from the Islet of Langerhans combined with omics’ approaches have led to detailed information of the transcriptomic environment of beta cells.

Gene-knockouts, and animal models, in particular targeting transcription factors combined with genome-wide association studies, have in the past decade provided us with a comprehensive map of genes and loci that may cause or contribute to diabetes, and it is evident that better understanding of molecular control(s) of functional beta cell mass will be key to design strategies to defeat diabetes.

All classical studies, not surprisingly, report a very high abundance of insulin mRNA, and insulin represents one of the most abundant cell specific mRNA’s known in our body. It was therefore surprising to realize that the second most abundant beta cell mRNA, recently reported in FACS purified human beta cells [1], was encoding INS-IGF2. Values comparable to glucagon mRNA levels in purified human alpha cells was reported for INS-IGF2 suggesting that a putative and highly abundant beta cell protein could have been overlooked. Moreover, recent studies suggested that INS-IGF2 could be yet another beta cell specific antigen recognized by autoantibodies in T1D [3].

## Results

### Transcriptome analysis

In our reanalysis of the RNA sequencing data from Nica et al. [1] we did not map the reads to transcripts but rather investigated an unbiased mapping of the reads to the genomic region on chromosome 11 containing the INS, IGF2 and INS-IGF2 transcripts. By visual inspection of the distribution of the reads (Fig. 1), it is clearly observed that the regions specific to INS-IGF2 are lowly expressed, whereas the INS specific exons are (as expected in beta cells) highly expressed.

Both coding exons of INS being expressed at the same order of magnitude is a clear indication that (a) most reads mapped to the exon that is shared by both INS and INS-IGF2 belong to the INS-001 transcript, and (b) the perceived high expression of the INS-IGF2 transcript is due to the read count being assigned to both genes, where the difference in level is due to the different lengths of the two genes.

The data analysis artefact is especially pronounced in this case, since INS in itself constitutes a large part of the transcript pool in beta cells. In our own experiments using the human beta cell line EndoC-βH1 we observe the same trends, both with RNA sequencing and micro-array analysis (unpublished, data not shown).

Both RNA sequencing and DNA micro-arrays are measuring sequence fragments, therefore we decided to take the analysis one step further and devised a qPCR strategy based on splice-form specific primers in order to provide a more direct measurement of the individual unfragmented mRNA species, in both EndoC-βH1 cells and human islets. In both cases the observations are the same; the INS-IGF2 fusion transcript is expressed at a level >20,000 fold lower than the INS transcript (see Table 1), and is hence barely detectable.

**Table 1:** qPCR analysis of INS-IGF2, INS and IGF2

Cell type	Gene	cDNA input (ng)	C_t_ values					GAPDH normalized exp. Rel. To INS
			Prep1	Prep2	Prep 3	Mean	GAPDH norm	
Human islets	INS	0.05	20.5	18.9	20.4	19.9	−6.3	1
Human islets	INS-IGF2	0.05	No C_t_	33.3	34.8	34.8	8.1	0.000046
Human islets	IGF2	0.05	32.8	32.7	31.3	32.3	6	0.000194
Human islets	GAPDH	0.05	26.9	27	24.9	26.2	0	–
EndoC-βH1	INS	5	14.8	14.3	14.4	14.5	−3	1
EndoC-βH1	INS-IGF2	5	29.9	30.8	28.3	29.7	12.2	0.000072
EndoC-βH1	IGF2	5	18.3	19.2	18.2	18.6	1.1	0.059678
EndoC-βH1	GAPDH	5	17.2	18.2	17.1	17.5	0	–

Relative expression of INS-IGF2, INS and IGF2 determined by qPCR for EndoC-βH1 cells and human islets. All mRNAs were analysed in 3 independent preparations, each in technical duplicate and the average of each duplicate is shown for each preparation (prep). GAPDH was used as input control. It is clear that INS-IGF2 is expressed at very low levels compared to Insulin, i.e. in the order of >20,000 fold less in human islets and 40,000 fold less in the human beta cell line EndoC-βH1.

### Proteome analysis

To finalise our conclusion that INS-IGF2 expression levels have been vastly overestimated, we investigated the abundance of the INS-IGF2 fusion protein using a strategy involving both immunochemical detection and mass spectrometry.

**Fig. 2:**
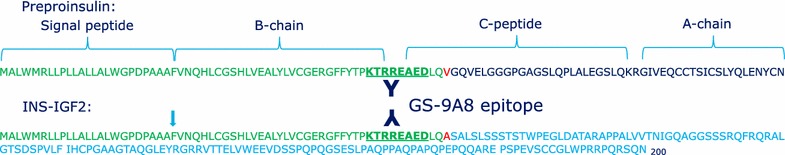
Protein level sequence comparison between unprocessed preproinsulin and the INS-IGF2 fusion protein. The epitope of the proinsulin specific monoclonal antibody GS-9A8 is indicated [4]. The BO1P antibody also used in this study was raised against the full length INS-IGF2 protein.

**Fig. 3:**
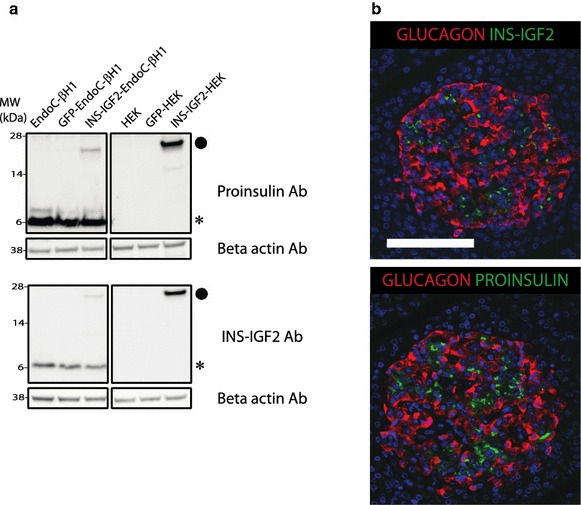
Immunoblot analysis and immunostaining of human pancreas. **a** Western blots investigating the presence of proinsulin-immunoreactivity (using antibody GS-9A8, *upper panel*) and INS-IGF2 fusion protein immunoreactivity (using antibody BO1P, *lower panel*) in the human beta cell line EndoC-βH1 and HEK, non transduced, GFP transduced or INS-IGF2 transduced. Endogenous proinsulin is marked with (*Asterisk*) and INS-IGF2 with (*filled circle*). Notice that INS-IGF2 is only reliably detected in cells transduced with INS-IGF2 construct. We titrated the levels of transduced HEK293 cell extract to give comparable band intensities on EndoC-βH1 proinsulin and HEK293-INS-IGF2 (*panel* **a** *upper*: GS-9A8 which has assumed identical affinities to the two proteins). INS-IGF2 transduction of EndoC-βH1 leads to relative lower expression of INS-IGF2-protein compared to proinsulin (*panel* **a** *upper* comparing the two bands in lane INS-IGF2-EndoC-βH1). This low-level expression of INS-IGF2 protein in transduced EndoC-βH1 is readily detected using the INS-IGF2 antibody while untransduced cells are completely negative (*panel* **a** *lower*). Thus we conclude that the expression of INS-IGF2 protein in EndoC-βH1 is below detection limits of this assay. **b** Immunoreactivity for INS-IGF2 (*green*) and glucagon (*red*) (*top panel*), and for proinsulin (*green*) and glucagon (*red*) (*bottom panel*) on adjacent sections of human pancreas. *Scale bar* 100 µM.

For the immuno-chemical work we used the only commercially available antibody (BO1P) raised against full-length INS-IGF2. This spans the preproinsulin signal-peptide, the entire B-chain of insulin and the first 8 amino acids of the C-peptide (which then continues into sequences that are unique for the INS-IGF2 protein—and thus not shared by IGF2 itself, see Fig. 2). As can be seen from Fig. 3, the commercial antibody readily detects the expression of recombinant INS-IFG2 in transduced HEK-293 cells and in transduced EndoC-βH1 cells. It is important to note that this antibody (also used by Kanatsuna et al. [3]) show strong cross-reaction to proinsulin in EndoC-βH1 cells whereas it only detects a band with the expected MW of INS-IGF2 in transduced cells (Fig. 3, panel a). We thus observe that the protein level of INS-IGF2 in EndoC-βH1 is below detection limit by Western blotting.

The control-antibody GS-9A8 (Madsen, Frank et al. [4]), a well characterized proinsulin-specific antibody binding a linear epitope spanning the B-C junction (Fig. 2) show full cross-reaction to recombinant INS-IGF2 (Fig. 3, panel a upper)—as would be expected with the intact proinsulin-B-C junction preserved in the INS-IGF2 protein (Fig. 2).

From band intensities in Western blotting experiments we conclude that the commercial polyclonal antibody BOA1 has higher affinity towards INS-IGF2 than for proinsulin and yet it fails to detect any INS-IGF2-like immunoreactivity in native human EndoC-βH1. In transduced EndoC-βH1 the expression of INS-IGF2 is much lower than proinsulin. Yet a weaker band is readily detected by BOA1 but completely absent in native human EndoC-βH1. Similarly, the GS-9A8 antibody having a presumed equal high affinity for both proinsulin and INS-IGF2 completely fails to detect an INS-IGF2 band in native human EndoC-βH1. In summary, these data are in line with the low levels of transcript we see in the human beta cell line, EndoC-βH1, which again raise the question do the protein exist?

We do observe that the antibody raised against INS-IGF2 (BO1P) indeed stain human beta cells on section of human pancreas (Fig. 3, panel b), but we cannot deduce if this is *only* due to the expected cross-reaction to proinsulin. In fact, the two antibodies, GS-9A8 and BO1P display very similar staining of proinsulin-like immuno-reactivity in human beta cells (Fig. 3, Panel b).

In order to further investigate the abundance of the INS-IGF2 protein level we conducted a comprehensive analysis of both EndoC-βH1 cells as well as isolated human islets using high-resolution mass spectrometry. Human islets were digested by trypsin and fractionated by either hydrophilic interaction chromatography (HILIC) or strong cation exchange (SCX) chromatography prior to reverse phase liquid chromatography tandem mass spectrometry (LC–MS). Our 2D fractionation method combined with high resolution MS allowed us to identify a total of 8,441 distinct human islet proteins. As expected, we were able to identify human insulin with five unique peptides and a high Mascot protein score (>500).

Moreover, we did identify proteins known to be expressed at a notoriously low level including transcription factors (e.g. FOS-B, NAC-2, SOX4 and SOX5), nuclear receptors (e.g. NR2F6 and NR5A2) and nuclear receptor coactivators (e.g. NCOA2, NCOA4 and NCOA6). However, even though we identified close to 8,500 proteins in our analysis we were not able to identify peptides specific to the INS-IGF2 protein in the samples. This indicates that INS-IGF2 is at most expressed in exceedingly low abundance in human islets.

In the proteomic analysis of the human beta cell line EndoC-βH1 we again identified a large number of distinct proteins (close to 7,000) including insulin and low abundant proteins but again, we were not able to identify peptides specific to the INS-IGF2 protein.

However, the caveat one has to keep in mind with MS-based proteomics experiments is the fact that absence of evidence is not evidence of absence. Therefore, in order to ensure that the INS-IFG2 protein could indeed be identified by our proteomics approach, HEK cells transduced with either INS-IGF2 or a control construct were subjected to the same analysis. Both samples were analysed by LC–MS and peptides specific for INS-IGF2 were readily observed only in the sample transduced with the INS-IGF2 construct. The missing evidence of INS-IGF2 protein in human islets and in EndoC-βH1 cells is therefore not related to our LC–MS approach.

In summary, based on sensitive proteomic and immunologic detection methods using adequate positive controls, we conclude that INS-IGF2 protein is expressed in exceedingly low abundance in normal human islet beta cells as well as in EndoC-βH1. This finding correlates well with the exceedingly low expression level of the INS-IGF2 mRNA.

## Discussion

We see the significance of the observations presented here to be two-fold:

1. To increase the awareness of the type of data interpretation artefacts fusion transcripts (or other overlapping sequence features) can inflict upon gene expression analysis for both RNA sequencing and micro-array experiments. In our opinion, the main problem is that the analysis artefacts can go unnoticed, which can lead to the publication of misleading results and wasted efforts in follow-up experimental work. In addition to the issue with the abundance of INS-IGF2 being over-reported, we have seen several published micro-array studies, where INS wrongly was reported to be missing on the array (with comments along the line of “*…Ins gene was excluded from ranking as it was not annotated in our Affymetrix chip…*” [5]).

The problem with over-reporting of overlapping transcripts may actually be more prevalent than immediately evident, as most cases are likely to be much less extreme than the case of insulin and INS-IGF2 in beta cells and thus go unnoticed. In fact, there are in total 459 human protein-coding transcripts that share exons in Ensembl 77, that could potentially be affected by the same misassignment as reported here.

On a more fundamental level, we recognize that biology is complicated and it is difficult to devise one true interpretation of the transcripts. Furthermore, a complicating factor is that as more and more transcripts variants are sequenced and deposited into the genomic databases (e.g. from large scale efforts such as the ENCODE project [6]), the risk of encountering this kind of problems is therefore likely to increase.

Part of this problem is, in essence, that these variants will carry the same weight in the analysis, even if some of them are exceedingly rare. On a more technical note, there is also the issue of what is classified as a gene in its own right as opposed to a transcript variant. In the case of INS-IGF2 part of the problem is that it’s listed as an independent gene and not as a splice variant of INS, as the analysis artefact could otherwise be side-stepped by aggregating the signal at the gene level.

While devising a “perfect” solution to the problem with overlapping genes is far from trivial, following an approach of listing which other transcripts may contribute to the signal of any given gene, could help to bring potential problems out in the open.

Lastly, it should be mentioned that several recent publications of islet transcriptomics data do not report on a faulty INS-IGF2 expression as they specifically handle this issue. Eizirik et al. [7] used the Flux Capacitor approach [8] to specifically handle the problem of reads mapping to exonic regions shared by multiple transcripts. Moran et al. [9] addressed the multiple transcript mapping issue by applying custom parameters in the RNA sequencing pipeline. The issue of handling exons from multiple transcripts is highly dependant on the software tool used, including its version and default parameters. For example, for users of the TopHat2 software package it should be noted, that the default settings for handling multiple transcript mapping was changed around September 2012 [10].

2. In the more specific case of INS-IGF2 it is important to avoid a chase for an elusive transcript for the many groups of scientists working with beta cells and islet biology. However, the big question remains on how important this transcript is? And does it indeed lead to the production of a novel beta cell specific protein? We show that the INS-IGF2 antibody as well as our proinsulin-specific antibody recognize an expected band of MW 22,000 in INS-IGF2 transduced cells (Hek293 and EndoC-βH1), as well as the native proinsulin band from the human beta cell line. The work of Kanatsuna et al. [3] concludes that INS-IGF2 represents a novel autoantigen in T1D, as they use in vitro translated/labeled INS-IGF2 to detect specific autoantibodies in T1D patients. However, “pre-proinsulin” autoantibodies would possibly also cross-react effectively in this assay, and such autoantibodies are well known in T1D. As an example, we show that proinsulin-B/C junction specific antibody fully cross-reacts to INS-IGF2. More specific reagents, such as antibodies raised against the unique c-terminal fragment of the predicted INS-IGF2 protein, are required in order to detect the protein specifically by immunocytochemical techniques.

Finally, our data failed to demonstrate the existence of the INS-IGF2 mRNA and protein in EndoC-βH1 cells. This was further supported by lack of detection of the protein by proteomics analysis, both in human islets and the beta cell line.

## Conclusions

Overlapping sequence features are problematic to handle in expression analysis pipelines. This challenge potentially leads to situations were some variants appears to be much higher expressed than data can support, or in the extreme case to completely mask out other genes in the vicinity. A large part of the problem is the “black box” nature of the gene expression analysis pipeline in the eyes of researchers not experts in bioinformatics, and that such errors may therefore go unnoticed. While the underlying mapping issue is far from trivial to solve, we suggest that by bringing the problem out in the open (e.g. by listing other factors contributing to the signal of a given gene), it will be easier to flag potential problems with a given data set, and to help avoid over-, or underestimation of gene expression levels.

Our in-depth analysis concludes that INS-IGF2 abundance is >20,000 times lower or undetectable at the level of mRNA and protein, respectively, compared to previous reported findings in human beta cells.

Finally, we suggest that pre-proinsulin antibodies (T1D autoantibodies) will also cross-react to recombinant INS-IGF2 protein, as exemplified by a proinsulin-B-C junction specific monoclonal antibody, GS9A8.

In conclusion, we suggest that more specific antibody reagents (towards against INS-IGF2 unique epitopes) need to be developed to facilitate and support investigations into whether INS-IGF2 fusion protein actually might be induced to elicit an autoantigen response in T1D.

## Methods

### Reanalysis of RNA sequencing data

For the re-analysis of the human Islet of Langerhans RNA sequencing data from Nica et al. [1], data was downloaded from the European Genome-phenome Archive [11] (accession: EGAS00001000442) as processed BAM files [12].

The BAM files follow the original analysis performed by Nica et al. [1]: briefly, the transcript-to-genome mapping was done using assembly version GRCh37/hg19 from February 2009 and GENCODE annotation v10 [13], which corresponds to Ensembl [14] v65. They further used BWA, Burrows-Wheeler Aligner [15], and filtered the alignments for correct orientation of the mapped mate pairs with a maximum insert size of 500 kb, and a minimum mapping quality score of 10.

In our re-analysis the processed BAM files were sorted and indexed and the relevant genomics region, chr11:2103768-2222439 (CGRh37/hg19 coordinates), was retrieved as Sequence Alignment/Map (SAM)-files via samtools [12]. Finally the read depth were calculated via samtools (v 0.1.19) and plotted using R (v. 3.0.2) [16]—we here observed that the read depth had been capped at 8,000. Exon/Intron coordinates for INS, IGF2 and INS-IGF2 transcripts were obtained from ENSEMBL [14] version 75 (also CGRh37/hg19 coordinates). See Additional file 1: Figure S1 for an in-depth overview of transcripts.

### Primer design

DNA sequences from the following transcripts were downloaded from ENSEMBL v. 75 [14]: INS-IGF2-001 (Ensembl: ENST00000397270), INS-001 (Ensembl: ENST00000381330), IGF2-001 (Ensembl: ENST00000416167). For primers uniquely targeting INS and INS-IGF2 coding exons the primer placement strategy shown in Additional file 1: Figure S1(panel B) was used, and following the verification of the unique parts of the transcripts using global pairwise alignment the primer sets listed below were designed. For the IGF2 coding exons there are no overlaps with other genes (see Fig. 1 and Additional file 1: Figure S1 panel A), and the probes were trivial to design.

Primerset 1 (INS specific), Forward: GCAGGTGGAGCTGGGC, Reverse: GGAGGAGAGGGACAAAGCTG. Primerset 2 (INS-IGF2 specific), Forward: CTACCTAGTGTGCGGGGAA, Reverse: ATTGTTCCACAATGCCACGC. Primerset 3 (IGF2 specific), Forward: GATGCTGGTGCTTCTCACCT, Reverse: CAGACGAACTGGAGGGTGTC. For normalization of cDNA input we used GAPDH, Forward: AGGGCTGCTTTTAACTCTGGT, Reverse: CCCCACTTGATTTTGGAGGGA. Primers were synthesized by Eurofins.

### qPCR

Quantitative RT PCR was carried out by standard methods. Briefly, RNA was purified with RNeasy (Qiagen) and checked on a Nanodrop instrument. All OD260/280 was ~2. The cDNA was made from 0.5 µg RNA in 20 µl iSCRIPT (Roche) reaction. For qPCR 2.5 µl of 10× (EndoC-βH1 cells) or 1,000× (Human islets) diluted cDNA was analysed with specific primers (see Table 1) and Brilliant II SYBR green mastermix (Agilent) using a Stratagene 3000p instrument. All PCR products were verified by melting curve analysis.

### Cell lines

The following cell lines were used in the study. *EndoC-βH1* Human pancreatic beta cell line (EndoC-βH1) [17]. The cells were grown as described in ref [17]. *HEK293* Human embryonic kidney cell line (ATCC CRL-1573). The HEK293 cells were grown in DMEM with 10% FBS and 1% P/S.

### Human pancreatic islets

Human islets were obtained via Prodo Laboratories Inc., CA, USA. Human pancreases were procured from cadaveric donors after written informed research consent was provided by donor relatives. All experiments were performed in agreement with national legislation and institutional ethical rules. Upon arrival, the islets were cultured in CMRL1066 with 5.6 mM glucose supplemented with 10% FCS, 2 mM l-Glutamine, 100 units/ml Penicillin and 100 μg/ml streptomycin at 37°C, under 95% air/5% CO2.

Human islet donor data: 3 donors, 1 female, 2 males; age, mean 34 years (range 30–38); BMI, mean 31.5 (range 28.5–33.1); purity, mean 90% (range 85–95); viability, mean 94.7 (range 94–95%). None of the donors were diagnosed with diabetes.

### Transduction

The INS-IGF2 cDNA sequence was obtained from GenBank [18] (accession id: NM_001042376.2) and the corresponding DNA was synthesized and inserted into a lentivirus vector (Gentarget Inc.). Lentivirus was produced in HEK293 cells and purified into PBS by ultracentrifugation. The MOI was 10 for EndoC-βH1 cells and 2 for HEK293 cells. Selection was done with puromycin for 1–3 weeks. As control we used the same backbone vector with a GFP cDNA insert.

### Immunohistochemistry

Human pancreas paraffin sections (Zyagen) were dewaxed and rehydrated to double-distilled H_2_O. Sections were microwave treated for 15 min in Tris-EGTA buffer (pH 9.0) and were allowed to cool for 30 min and rinsed in double-distilled H_2_O. Slides were blocked with TNB blocking buffer (Perkin Elmer) for 30 min, followed by incubation with the primary antibody guinea pig anti Glucagon (1:500; Linco) in combination with the mouse monoclonal anti proinsulin clone GS9A8 (1:100; Developmental Studies Hybridoma Bank), or the mouse polyclonal anti INS-IGF2 (1:100; BO1P Abnova) diluted in TNB o.n at room temperature. Three washes in PBS were then followed by incubation with fluorescent labelled secondary antibodies (Cy2 or Cy3 conjugated donkey anti guinea pig and anti rabbit IgG (1:400; Jackson ImmunoResearch)) and DAPI diluted in TNB and three washes in PBS before mounting. Images were acquired using confocal microscopy (Zeiss LSM 510).

### Western blot

Cultured HEK293 or EndoC-βH1 cells were harvested in Cell Extraction Buffer (Life Technologies) freshly supplemented with Complete Protease Inhibitor Cocktail (Roche). Samples were run on 12% NuPAGE SDS gels, under reducing conditions in MES-SDS buffer (Invitrogen) and then transferred on PVDF membrane for chemiluminescent Western blot. Anti-proinsulin and INS-IGF2 antibodies have been used at 1:1,000 dilution, monoclonal anti Beta actin-peroxidase (Sigma) at 1:25,000. Secondary antibody goat anti mouse IgG (Santa Cruz Biotechnology) was diluted 1:2,000.

### Mass spectrometry

Human islets or EndoC-βH1 cells were lysed in 0.5% RapiGest (Waters, Milford, MA, USA) dissolved in 50 mM ammonium bicarbonate. The islets were subsequently reduced in DTT (10 mM) and alkylated with 45 mM chloroacetamide. The sample was digested with trypsin (1:50 w/w) O.N. at 37 degrees. To precipitate the RapiGest (Waters, Milford, MA, USA) 10% TFA was added to a final concentration of 0.5%. The peptides were centrifuged for 20 min at room temperature and purified in a SepPak (50 mg) cartridge (Waters, Milford, MA, USA). The peptides were eluted with 50% acetonitrile and lyophilized. For HILIC fractionation, the peptides were dissolved in 4 ul buffer A (0.1% TFA) and 36 ul buffer B (98% acetonitrile, 0.1% TFA). The peptides were fractionated using a gradient of 55 min (95–60% B). The fractionation was done using an Agilent uHPLC 1290 system (Agilent Technologies, Santa Clara, USA) For SCX fractionation, the column (StageTips with 3 M Empore SCX disks, 3 M Empore, MN, USA) was conditioned with (1) 100% acetonitrile, (2) 50% acetonitrile pH4.0, (3) 50% acetonitrile pH 11, (4) 40% acetonitrile with 0.1% TFA. The peptides were loaded in 40% acetonitrile with 0.1% TFA and subsequently step eluted using a buffer containing 20 mM acetic acid, 20 mM boric acid, 20 mM phosphoric acid, 50% acetonitrile with pH adjusted to 4.0; 4.5; 5.0; 6.5; 7.5; 11.0, respectively. Peptides from HILIC and SCX fractionation were analysed by LC–MS using an Easy-nLC 1000 system coupled to a Q-Exactive Orbitrap mass spectrometer (Thermo Fisher Scientific, Bremen, Germany). The peptides were separated on a 90 min gradient (8–40% acetonitrile in 0.1% formic acid) on a reverse-phase column (1.9 µm beads, Dr Maisch GmbH, Ammerbach, Germany). The raw data was searched using the Mascot search engine in Proteome Discoverer v1.4 (Thermo Scientific, Bremen, Germany) with a peptide false discovery rate of less than 1%, which was estimated on the number of reverse hits. Carbamidomethylation and oxidation of methionine were specified as variable modifications.

## Additional file

Additional file 1:
**Figure S1.** A) All ENSEMBL transcripts defined in the INS/IGF2 region; B) Placement stragety for INS and INS-IGF2 specific qPCR primers.
