# Supplementary material for: Analysis artefacts of the INS-IGF2 fusion transcript
Source: BMC Mol Biol. 2015 Jul 29;16:13. doi: 10.1186/s12867-015-0042-8 (PMC4517550; doi:10.1186/s12867-015-0042-8)
Supplement: Additional file 1: — Figure S1. A) All ENSEMBL transcripts defined in the INS/IGF2 region; B) Placement stragety for INS and INS-IGF2 specific qPCR primers. [file 12867_2015_42_MOESM1_ESM.docx]

# SUPPLEMENTARY material to:

**Analysis artefacts of the INS-IGF2 fusion transcript.**

### Rasmus Wernersson, Thomas Frogne, Claude Rescan, Lena Hansson, Christine Bruun, Mads Grønborg, Jan Nygaard Jensen, Ole Dragsbæk Madsen

# Figures

## Figure S1:

Full view of transcripts defined by ENSEMBL[1] in the INS / IGF2 region on chromosome 11 (image source: ENSEMBL v. 75 – coordinates: 11:2151,500-2183,000). **Panel A:** full overview of the genomic region (notice that the transcripts being investigated are all are encoded on the reverse strand). **Panel B:** Zoom of the INS, INS-IGF2 region, and conceptual placements of the transcript specific qPCR primers.

# References

1. Flicek P, Amode MR, Barrell D, Beal K, Billis K, Brent S, Carvalho-Silva D, Clapham P, Coates G, Fitzgerald S, Gil L, Girón CG, Gordon L, Hourlier T, Hunt S, Johnson N, Juettemann T, Kähäri AK, Keenan S, Kulesha E, Martin FJ, Maurel T, McLaren WM, Murphy DN, Nag R, Overduin B, Pignatelli M, Pritchard B, Pritchard E, Riat HS, et al.: **Ensembl 2014**. *Nucleic Acids Res* 2014, **42**:D749–D755.
